# Comparing upper limb motor recovery in subacute ischaemic stroke and intracerebral haemorrhage: A Systematic Review.

**DOI:** 10.12688/healthopenres.13450.2

**Published:** 2025-05-06

**Authors:** Lara Grima, Sally Davenport, Adrian R. Parry-Jones, Andy Vail, Ulrike Hammerbeck

**Affiliations:** 1 Physiotherapy Group, GOSICH, University College London, London, England, WC1N 1EH, UK; 2 Division of Cardiovascular Sciences, The University of Manchester, Manchester, England, M13 9PL, UK; 3 Geoffrey Jefferson Brain Research Centre, The University of Manchester, Manchester, England, M6 8HD, UK; 4 Centre for Clinical Neurosciences, The University of Manchester, Manchester, England, M6 8HD, UK; 5 Centre for Biostatistics, The University of Manchester, Manchester, England, M13 9PL, UK; 6 Centre for Human and Applied Physiological Sciences, King's College London, London, England, SE1 1UL, UK

**Keywords:** Ischaemic stroke, Intracerebral haemorrhage, motor recovery, upper limb

## Abstract

**Background:**

The pathophysiology and medical management between ischaemic stroke and intracerebral haemorrhage differ as do their functional independence and mortality outcomes. This paper aims to establish whether their respective upper limb motor impairment and recovery differ. This information could inform discussions with patients about their recovery prognosis as well as identify appropriate rehabilitation settings.

**Methods:**

A PROSPERO registered systematic search of three databases (MEDLINE, CINAHL, Embase) identified studies that measured upper limb motor function (Fugl-Meyer assessment scale for upper extremity) in participants with first stroke (ischaemic stroke or intracerebral haemorrhage) within 31 days post-stroke and at least one follow-up assessment. Risk of bias was assessed using the Critical Appraisal Skills Programme.

**Results:**

The search identified 1108 studies of which three met inclusion criteria, with a total of 258 participants (200 ischaemic stroke, 58 intracerebral haemorrhage). All studies had low to moderate risk of bias. At baseline, participants with intracerebral haemorrhage had greater upper limb motor impairment on the Fugl-Meyer assessment scale, but at six months post-stroke, the stroke subtypes reached similar upper limb motor function. Improvements were greatest early after stroke.

**Conclusions:**

Despite greater severity at baseline, intracerebral haemorrhage survivors appeared to reach the same level of arm function at six months post stroke. However, these findings need to be interpreted with caution due to limited studies and small number of participants included in this review and warrant further research.

**PROSPERO registration:**

CRD42020159110 (19/02/2020).

## Plain Language Summary

Is arm recovery different between a stroke caused by a clot or a bleed? Clinicians make decisions of care pathways and medical treatment for patients early after stroke by what they expect their recovery will be. Most people have a stroke caused by a clot and a lot of research has been done about their recovery. But we don't know if recovery is the same in people who had a stroke caused by a bleed, rather than a clot. The purpose of this study is to see if previous studies can show if arm recovery is the same between these stroke types. We investigated arm recovery because it is the most common difficulty after stroke. We performed a literature search to find studies that measured arm recovery using the Fugl-Meyer upper limb assessment in both stroke types, from before 31 days after stroke and performed at least one follow-up assessment. We identified 3 studies with a total of 258 participants (200 individuals who had a stroke cause by a clot and 58 a stroke caused by a bleed). At the first assessment individuals with a stroke caused by a bleed had more arm weakness but at six month the arm weakness was very similar between the stroke types. Athough individuals with a stroke caused by a clot have more arm weakness early after stroke, they appear to recovery more. However, further studies need to investigate differences in recovery of arm function between these stroke types because our investigation only included a small number of studies.

## Introduction

Stroke places a significant burden on society and is the third-leading cause of death and disability globally ^1, 2^. Despite Intracerebral Haemorrhage (ICH) being globally far less common than ischaemic stroke (27.9% vs 62.4%), it accounts for a greater proportion of the global burden ^2^. The burden of stroke can be attributed to many factors, with the most common impairment after stroke being upper limb motor weakness ^3, 4^, from which recovery is poor ^5, 6^. Arm weakness impacts on stroke survivors’ independence, quality of life and ability to maintain previous life roles ^7, 8^. Despite significant recent advances in stroke care, over 20% *of* stroke survivors do not recover functional use of their arm ^3^. The impact of poor arm recovery is considerable with an estimated 100,000 strokes occurring in the UK every year, and 1.2 million stroke survivors alive at any time ^9^. However, longitudinal epidemiological studies of stroke recovery are very rare ^10^ and the natural history of recovery after stroke not well understood, especially in ICH. Arm recovery is influenced by patient demographics, stroke severity and acute care ^11, 12^. However, it is currently unclear whether upper limb impairment and recovery differs between ICH and ischaemic stroke because stroke recovery tends to be reported without differentiating between stroke types. 

The pathophysiology of ischaemic stroke and intracerebral haemorrhage is fundamentally different. Ischaemic stroke is caused by an occlusion in a cerebral blood vessel ^13^ with the ischaemia-related cascade causing alterations in the function of glial cells and neurons ^14^. In contrast, ICH is caused by the rupture of a blood vessel with resultant blood in brain tissue. Therefore, in addition to ischaemia, the pathophysiology of ICH involves the mass effect of the haematoma on cerebral tissue ^15, 16^, and the interaction between components of the haematoma and the brain ^17^. In light of these pathophysiological differences, it is likely that upper limb motor recovery differs with regards to the extent as well as timeline for these stroke subtypes.

An understanding of recovery differences in upper limb motor function between ICH and ischaemic stroke will inform discussions about recovery prognosis between clinicians and patients. The knowledge will shape goal setting and discharge planning. Furthermore, this information could assist clinical decision-making with regards to life saving interventions early after stroke and rehabilitation pathways at later time points. Currently, negativity of ICH outcome prevails, and clinicians do not instigate lifesaving interventions including admission to intensive care and neurosurgical evacuation of the haematoma when patients with ICH present with severe impairments at baseline ^18^. We here aim to establish whether there is evidence of differences in upper limb motor recovery between ischaemic stroke and ICH by performing a systematic review of the literature.

## Methods

This review was reported according to PRISMA guidelines (Supplementary Table 1 ^19^) and was registered prospectively with PROSPERO (CRD42020159110) on 19 ^th^ February 2020.

### Patient and Public Involvement

There was no Patient and Public Involvement in the delivery of this systematic review.

### Eligibility criteria

We included observational, randomised and non-randomised controlled trials (RCT) conducted in adults with stroke. Studies were included if they recruited at least 15 participants each with ischaemic stroke and ICH. Smaller studies would increase the risk of a type-2 error. Studies needed to measure upper limb motor function using the Fugl-Meyer Assessment Upper Extremity Subscale (FMA-UE) within 31 days of stroke onset (baseline) in addition to follow-up measures. Data had to be reported separately for stroke subtypes. Studies were excluded if (i) participants with previous stroke could not be excluded from the dataset, (ii) stroke subtype was not reported and (iii) raw data was not provided to address the above requirements. For RCTs, data from the treatment arm were excluded if there was a significant statistical difference between groups.

### Search strategy and study selection

A systematic search was conducted in Medline, Embase and CINAHL on the 17 ^th^ of December 2020. Search strings were based on concepts of ‘stroke’, ‘early,’ ‘motor recovery’, and ‘upper limb’. Searches were limited to studies published in English from 2000 to 2020, coinciding with the start of stroke units ^20^ (Supplementary Table 2 ^21^). The titles, abstracts and full texts were consecutively screened independently by two reviewers (LG and UH). Disagreement regarding inclusion was resolved through discussion. In addition, the references from key included publications were hand-searched for completeness of inclusion. Raw data were requested from authors in cases of 1) inclusion of participants with previous stroke, 2) some participants with baseline data after 31 days post-stroke or 3) if outcomes were not reported separately for ischaemic stroke and ICH patients.

### Data extraction

Data were extracted (LG) to Microsoft Excel and checked thoroughly (UH). When datasets (containing individual patient data) were obtained, participants with previous stroke and participants without baseline data before 31 days post-stroke were excluded. The data sought was upper limb motor function measured using the Fugl-Meyer Assessment Scale for the Upper Extremity (FMA-UE) at timepoints up to one year post-stroke. Baseline timepoints were at less than seven days and 3–4 weeks after stroke. Follow-up timepoints at which data were collected were at ten days, three weeks and one, three, six and twelve months after stroke.

### Risk of bias assessment

Study quality was assessed by two reviewers using the randomised control trial (RCT) and cohort studies Critical Appraisal Skills Programme (CASP) checklists respectively. We tabulated attrition observed in the three studies (Supplementary Table 3). In addition to documenting the missing numbers at each time point for the two stroke types, we compared their baseline impairment. To established whether attrition caused a risk of bias through missingness of more severely affected individuals. To do this we calculated the mean FMA-UE score for the missing participants at baseline and subtracted the mean FMA-UE baseline score for the stroke type in the study. Thereby a negative score would indicate that more severe individuals were missing for followed-up for the respective stroke type and highlight bias.

### Data synthesis and analysis

Descriptive statistics, using SPSS version 27, were used to calculate data separately for ischaemic stroke and ICH. Statistical tests to establish differences in baseline characteristics and outcomes between stroke types used independent samples t-test for normally distributed data, Mann Whitney U-test for non-normal data and Pearson’s chi-square test to investigate categorical distribution. Mean outcome measure scores at the respective time point for each stroke subtype and in each study were plotted on a chart. Trajectories of upper limb motor recovery of the two groups were compared.

As one study excluded the reflex measurement component from the FMA-UE ^22^, the outcome measure was converted to a percentage of the respective maximum score (66 or 60) and are represented as this throughout.

## Results

### Study identification

**Figure 1.**
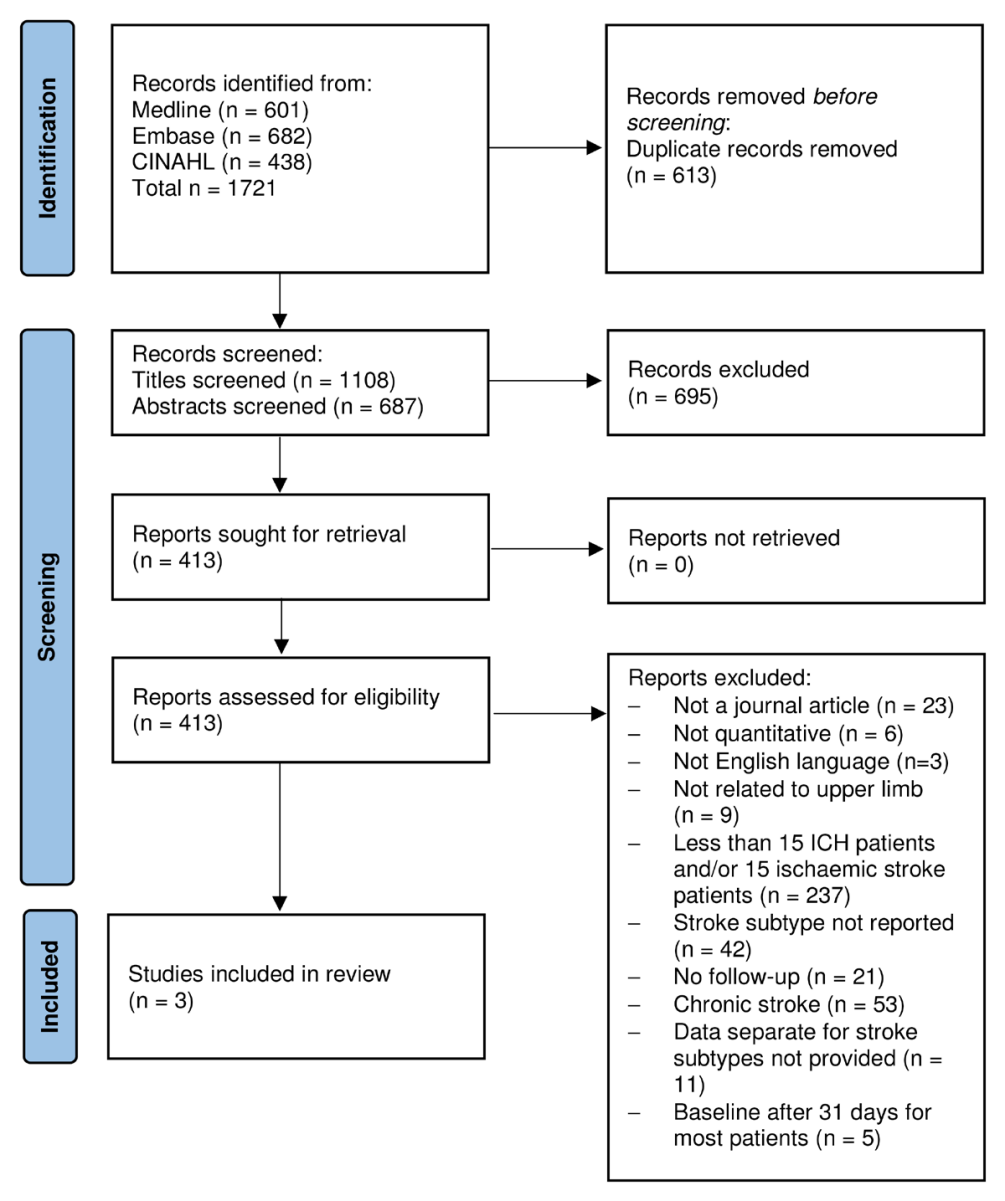
PRISMA flow diagram.

The search yielded 1108 studies after removal of duplicates ( Figure 1, PRISMA Flow Chart). After title and abstract screen, 413 full texts were screened, of which, 393 were excluded. Of the remaining 20 studies, one study reported outcome measures separately for stroke type and 19 reported that they included both IS and ICH survivors but did not report separate outcomes. Eligibility of these studies was dependent on the provision of separate outcomes for ischaemic stroke and ICH or raw data. Data from seven from the above 19 studies were obtained, but five studies were excluded because they did not satisfy all eligibility criteria, resulting in the inclusion of three studies in the review.

Data for ischaemic stroke and ICH baseline characteristics and outcome measure scores were collated ( Table 1 and Supplementary Table 4). 

**Table 1.** Included study details and baseline characteristics of participants.

		Ghaziani *et al.*, 2018 ^25^		Persson *et al.*, 2016 ^26^		Plantin *et al.*, 2019 ^22^	
**Study type**		RCT		Cohort		Cohort	
**Country**		Denmark		Sweden		Sweden	
**n**		80		117		61	
**Baseline post stroke**		<7 days		3 days		3–4 weeks	
Subtype		IS	ICH	IS	ICH	IS	ICH
**n (%)**		61 (76%)	19 (24%)	98 (84%)	19 (28%)	41 (67%)	20 (33%)
**Gender**	**Male** **Female**	33 (54%) 28 (46%)	7 (37%) 12 (63%)	56 (57%) 42 (43%)	9 (47%) 10 (53%)	27 (66%) 14 (34%)	14 (70%) 6 (30%)
		x ^2^_(80)_=1.73; p=0.189		x ^2^_(117)_=0.616; p=0.433		x ^2^_(61)_=0.106; p=0.746	
**Age, years** **mean (SD)**		72 (11)	70 (15)	71 (13)	63(10)	54 (2 ^&^)	50 (2 ^&^)
		t _(23.87)_=0.441; p=0.663		t _(115)_=2.495; p=0.014 *		t _(37)_=1.128; p=0.266	
**Stroke severity** **mean (SD)**		45 (9)	37 (11)	8 (6)	10 (6)	8 (1 ^&^)	8 (1 ^&^)
		SSS U _(80)_= 805.5; p=0.011 *		NIHSS U _(115)_=675; p=0.073		NIHSS t _(37)_=0.01; p=0.999	
**FMA-UE baseline** **mean SD**		37(19)/66	27(21)/66	30(25)/66	20(22)/66	26(4 ^&^)/60	22(6 ^&^)/60
**FMA-UE baseline normalised (%)**		56(28.9)%	41(31.3)%	45(38.1)%	30(32.6)%	43(6.7 ^&^)%	37(10 ^&^)%
**Stroke location**	**RH** **LH** **Bilateral** **Cerebellum** **Brainstem**	28 (46%) 33 (54%)	8 (42%) 11 (58%)	49 (50) 44 (45%) 4 (4%) 1 (1%)	11 (58%) 7 (37%) 1 (5%)	27 (66%) 14 (34%)	10 (50%) 10 (50%)

***Key:*** RCT: randomised controlled trial, FMA-UE: Fugl-Meyer Assessment for upper extremity, IS: ischaemic stroke, ICH: intracerebral haemorrhage, n: number of participants, SD: standard deviation, SSS: Scandinavian Stroke Scale (higher score means lower stroke severity), NIHSS: National Institutes of Health Stroke Scale (higher score means higher stroke severity), RH: right hemisphere, LH: left hemisphere, * statistical significance p<0.05, ^&^ measures likely to represent the standard error of the mean (rather than SD).

### Risk of bias

The risk of bias (Supplementary Table 4 and 5 ^23, 24^) in the reported studies was moderate to low ^25^ and moderate ^22, 26^ for the respective studies. This was largely due to the fact that important established confounding factors had not been identified or accounted for 22, 26. Furthermore, baseline measures of impairments were not matched and the study groups did not receive the same level of care ^25^. Attrition bias was observed (Supplementary Table 3 ^27^) but this was not greater in the ICH group than the IS group. Instead, there were more IS patients that were lost to follow-up and the patients lost to follow-up were more severely affected than the mean sample in IS than ICH. Therefore, the difference between the groups that we observed was not over-estimated, if anything attrition could have led to the improvements in the ischaemic stroke group being over-estimated.

### Characteristics of the studies and participants

The studies comprised a RCT and two cohort studies, which were all conducted in Scandinavian countries ( Table 1). The sample sizes were small comprising 61, 80 and 117 stroke survivors ^22, 25, 26^ with smaller percentages of ICH than ischaemic stroke participants (20, 24 and 28% respectively). Although there were more male participants overall, the gender distribution did not differ between stroke subtypes. In general, the groups were well matched except in one study where stroke severity was significantly greater ^25^ and in another study where age was significantly lower ^26^ in the ICH group. Baseline measurement was performed either within the first week ^25, 26^ or at three to four weeks post-stroke ^22^.

### Upper limb motor function

**Figure 2.**
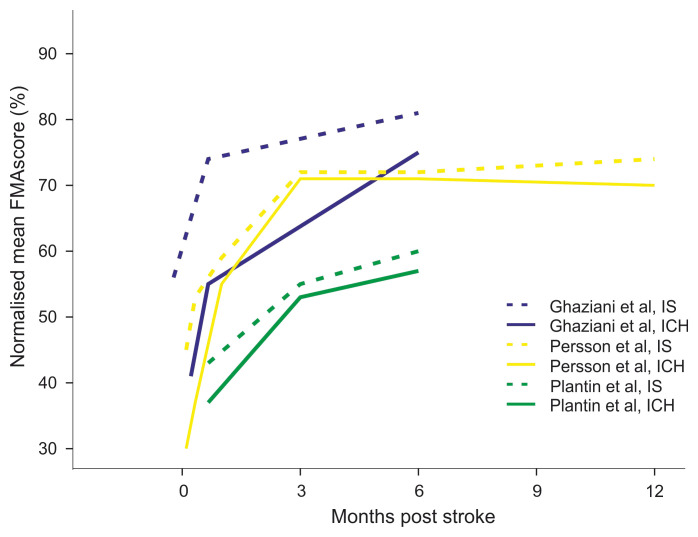
Comparison of longitudinal changes of the normalised FMA-UE score between ICH and IS survivors. Blue: Ghaziani *et al*. 2018, yellow: Persson *et al*. 2016, green: Plantin *et al*. 2019, continuous line: ICH, dotted line: ischaemic stroke.

Upper limb motor function at baseline, measured by the normalised FMA-UE score, was lower in all studies for ICH survivors ( Figure 2, Supplementary Table 6 ^28^) by between 4–10 points on the FMA-UE ( Table 1). In effect the mean scores for ICH were lower than any mean IS scores ( Figure 2). Over time, the FMA-UE score increased in all studies for both stroke types. At 6-month follow-up the difference between the groups had substantially reduced and was between less than 1 to 4 points of the FMA-UE (Supplementary Table 7 ^29^).

## Discussion

This systematic review identified three studies with data that allowed an exploration of differences in upper limb motor recovery using the FMA-UE between ICH and ischaemic stroke in the subacute phase after stroke. The studies had low to moderate risk of bias and included 258 participants with stroke (ischaemic stroke n=200, ICH n=58). We found that recovery was greater in ICH than ischaemic stroke. This related to individuals with ICH having more severe motor impairment at baseline but at 6-month follow-up, motor impairment was more similar to ischaemic stroke patients. The limited number of studies, low number of included participants and variability in the datasets/papers make firm conclusions impossible. However, these findings echo clinicians' perceptions of worse baseline motor impairment of ICH survivors with subsequent greater recovery and warrant further exploration.

Our data confirm that most recovery occurs early after stroke ^30^ both in ischaemic stroke and ICH. From evidence in animal models, these early improvements are largely attributed to spontaneous recovery mediated by altered brain activity on a molecular and cellular basis in response to injury ^30– 32^. Underlying processes for the recovery are thought to include the salvation of brain cells within the penumbra ^33^ and unmasking of latent synaptic pathways ^34^. However, these findings are largely based on ischaemic stroke models. We observed greater baseline motor impairment in ICH survivors in our dataset, which can probably be attributed to the additional pathological processes in ICH ^15, 16^. Specifically, these processes include mass effect from the space occupying haematoma as well as the detrimental interaction between blood and brain parenchyma early after insult ^16^. However, over the first two weeks, the mass effect reduces, and the haematoma resolves within 3 months ^17, 35^. We found that at six months post-stroke, the stroke subtypes had similar levels of upper limb motor function. After the subacute period, both ICH and ischaemic stroke survivors are essentially left with the damage caused by hypoperfusion of brain tissue ^34^.

At baseline, there were differences between ICH and ischaemic stroke survivors FMA-UE score. In all studies ICH survivors’ mean FMA-UE score categorised them as having severe impairment (<28) ^36^, whereas this was only the case in one study for ischaemic stroke survivors ^22^. The difference in arm impairment severity between ICH and IS survivors was in excess of the minimal clinically important difference in two studies ^37^.

The question of interest is however, the amount and pattern of recovery, and therefore the extent and nature of change ^38^. A commonly used model to understand the relationship between baseline severity and recovery is the proportional recovery rule ^39^. It proposes that recovery scales with severity and that 70% of stroke survivors will regain 70% of their lost motor function. Thereby, individuals with more impairment are postulated to have greater numerical improvement on the FMA-UE. However, this is not the case for the most severely affected stroke survivors, who tend to fall into the 30% of survivors who do not follow the proportional rule and do not recovery arm function. Recovery of ICH survivors in this small dataset appears not to comply with the rule. The rule is violated in two way. Firstly it appears as though individuals with ICH appear to recover more than those with ischaemic stroke since they started from lower levels of upper limb motor function. In addition, individuals with severe impairment do recover. Whether the recovery in the ICH group exceeds the 70% of lost function, as proposed in the Proportional Recovery Rule ^39^, is not clear in our small sample. Future work should establish whether recovery differs between ICH and ischaemic stroke in a cohort matched for baseline severity.

In this systematic review, we compared recovery of impairment, measured by the upper extremity component of the Fugl-Meyer Assessment. The FMA-UE was designed to measure stages of arm recovery, from severe paralysis to motor control without the use of stereotypical synergy patterns ^40, 41^. Recovery of other impairments and limitations of activity could therefore differ. However, in previous work recovery has been found to be remarkably similar regardless of whether it is measured by the FMA-UE, the Motricity Index (strength) ^42, 43^ or the ARAT ^44^ which measures limitations in activity. 

These findings do need to be interpreted with caution as the review has some limitations. Spontaneous recovery is the greatest early after stroke ^32^ but the included studies measured baseline FMA-UE, at up to three ^26^, seven ^25^ and 31 days ^22^ post stroke. It is therefore highly likely that some recovery had already occurred in the participants in that specific study ^22^. Rather than changing the findings this could have diminished the magnitude of the differences in baseline measures and the change we observed between the stroke types. In addition, the impact of high attrition needs to be considered when interpreting the findings. In these studies missing data often constituted a missed follow-up appointment, rather than dropping out of the study. Attrition bias is evident in ischaemic stroke survivors in our database (Supplementary table 3), as missed data was observed in individuals with predominantly poor upper limb motor function at baseline. This resulted in artificially high mean improvement in the ischaemic stroke subgroup. In our dataset, this does not appear to apply to the group with ICH, since very few patients did not attend for follow-up assessments and those who did not, were not consistently more severely impaired.

Considering that this review analysed data of only 58 participants with ICH, and found a high attrition bias in the IS group, further research is required to answer the research question with more confidence. A number of factors influence recovery after stroke including age, acute care and stroke severity which are difficult to control for in small studies. Conclusive evidence of recovery differences between stroke types would be important because in addition to informing patient and carer conversations about prognosis they are vital to inform appropriate care decisions. ICH survivors are far more likely to be placed on a palliative care pathway than ischaemic stroke survivors, irrespective of their baseline severity or other prognostic factors ^18^. Thereby they miss out on lifesaving early interventions including intensive care, neurosurgical interventions and specialist rehabilitation to optimise recovery. Evidence of the recovery potential of ICH survivors should be integrated into prognostic models that inform early medical decision making.

## Conclusion

In the limited studies that compare arm impairment and recovery in ischaemic stroke and ICH, baseline arm impairment was more severe in ICH. Despite this, the ICH survivors recovered arm movement to a similar level at six months after stroke. This indicates that there are differences in the recovery between these stroke types. 

## Data Availability

All data underlying the results are available as part of the article and no additional source data are required. Zenodo: Table 2: Search Strategies. https://doi.org/10.5281/zenodo.10079312 ^21^. Zenodo: Table 3: Attrition for IS and ICH patients in the three studies and comparison of FM baseline mean between missing participants and sample mean. https://doi.org/10.5281/zenodo.10059673 ^27^. Zenodo: Table 4: Quality appraisal results using the Critical Appraisal Skills Programme RCT checklist. https://doi.org/10.5281/zenodo.10059677 ^23^. Zenodo: Table 5: Quality appraisal results using the Critical Appraisal Skills Programme Cohort Studies checklist. https://doi.org/10.5281/zenodo.10059684 ^24^. Zenodo: Table 6: Normalised FMA-UE scores at different timepoints, according to study and stroke subtype. https://doi.org/10.5281/zenodo.10059689 ^28^. Zenodo: Table 7: FMA-UE scores at different timepoints, according to study and stroke subtype. https://doi.org/10.5281/zenodo.10059695 ^29^. Zenodo: PRISMA checklist for ‘Comparing motor recovery in ischaemic stroke and intracerebral haemorrhage: A systematic review’. https://doi.org/10.5281/zenodo.10059192 ^19^. Data are available under the terms of the Creative Commons Attribution 4.0 International license (CC-BY 4.0).
