# Supplementary material for: Comparing upper limb motor recovery in subacute ischaemic stroke and intracerebral haemorrhage: A Systematic Review
Source: Health Open Res. 2025 May 6;5:33. Originally published 2023 Nov 17. [Version 2] doi: 10.12688/healthopenres.13450.2 (PMC12134863; doi:10.12688/healthopenres.13450.2)
Supplement: Peer review history

# Reviewer report: Reviewer response for version 2

**DOI:** 10.21956/healthopenres.14923.r29432

**Published:** 2025-06-02

**Authors:** Salman Ikramuddin

**Affiliations:** 1 University Texas Health Sciences Houston, Houston, TX, USA

The authors set out to address an important and under appreciated area in the literature. Namely, a description of recovery trajectories comparing IS and ICH. While the concept is strong it is notable that only 3 studies directed provided the data they needed resulting in a small N compared to the existing data (just 58 with ICH). Of the 3 studies, there is some important heterogeneity including one with median age in the 50s, and another with much higher stroke severity. These issues make it difficult for the authors to answer the question they seek to address. If done well, this would be a well cited manuscript and inform recovery trials in the future. Current strengths of the manuscript include the concept, and a particularly strong discussion that puts the findings into context. 

 Major Comments

 1. It is not entirely clear what search terms were used, it would be reasonable to include in exact terms what booleans were used with the full date ranges (including month, day, year) for each search tool (medline, etc) in the main text of the manuscript. 

 2. The number of excluded studies is very high, it may be worthwhile to reach out to the first authors of excluded studies to inquire if the data separated out by stroke subtype is available. 

 Minor Comments

 1. Introduction paragraph 1: the rate of hemorrhagic stroke reported here seems higher than those typically cited, I did not clearly see it laid out in citation#2 where this number comes from. I would revisit this. 

 2. Discussion paragraph 4: typo, "The rule is violated in two way **s** ."

Are the rationale for, and objectives of, the Systematic Review clearly stated?

Yes

Is the statistical analysis and its interpretation appropriate?

Yes

Are sufficient details of the methods and analysis provided to allow replication by others?

Partly

Are the conclusions drawn adequately supported by the results presented in the review?

Partly

Reviewer Expertise:

Stroke Recovery

I confirm that I have read this submission and believe that I have an appropriate level of expertise to confirm that it is of an acceptable scientific standard, however I have significant reservations, as outlined above.

# Reviewer report: Reviewer response for version 2

**DOI:** 10.21956/healthopenres.14923.r29399

**Published:** 2025-05-07

**Authors:** Margit Alt Murphy

**Affiliations:** 1 Clinical Neuroscience, University of Gothenburg, Gothenburg, Västra Götaland County, Sweden; 2 Sahlgrenska University Hospital, Gothenburg, Sweden

I dont have any more questions.

Are the rationale for, and objectives of, the Systematic Review clearly stated?

Yes

Is the statistical analysis and its interpretation appropriate?

Yes

Are sufficient details of the methods and analysis provided to allow replication by others?

Yes

Are the conclusions drawn adequately supported by the results presented in the review?

Yes

Reviewer Expertise:

Upper limb motor rehabilitation in stroke

I confirm that I have read this submission and believe that I have an appropriate level of expertise to confirm that it is of an acceptable scientific standard.

# Reviewer report: Reviewer response for version 1

**DOI:** 10.21956/healthopenres.14537.r27658

**Published:** 2024-01-03

**Authors:** Pradeepa Nayak

**Affiliations:** 1 Manchester Metropolitan University, Manchester, England, UK

The manuscript addresses a significant gap in the literature and is well written. This systematic review follows PRISMA guidelines and summarises the literature on motor recovery in ischemic stroke and intracerebral haemorrhage. There is logical reporting throughout the manuscript which makes it easy to read. To enhance clarity, some components of the manuscript could be further expanded.

 Title reads as: Comparing motor recovery in ischaemic stroke and

 intracerebral haemorrhage: A Systematic Review. In the title, authors might want to be specific and consider the addition of ‘upper limb’ motor recovery and stage of stroke recovery of the chosen population (acute and subacute), as well as the chosen outcome measure (FMA UE), as the review focused only on upper extremity motor recovery and is very specific to the outcome measure used i.e.  FMA (other outcome measures of motor recovery were not included) and focused on acute and subacute stroke (excluded adults with chronic stroke).

 Abstract: It might be good to add aim/objective of the review under the ‘background’ than stating the implications of the research. This line can be replaced with the aim of the review - “This information could inform discussions with patients about their recovery prognosis as well as identify appropriate rehabilitation settings.”

 Introduction: Introduction looks brief. It would be good to give some background information/ literature available on the upper limb recovery in people with ischaemic and intracerebral haemorrhagic stroke under introduction. It is good to state the aim of the review in the last part of the introduction.

 It’s unclear how the following statement relates with the current study, as the current study examines the motor recovery post stroke. Authors might want to link the statement with the present study with the use of evidence - “Currently, negativity of ICH outcome prevails, and clinicians do not instigate lifesaving interventions including admission to intensive care and neurosurgical evacuation of the haematoma when patients with ICH present with severe impairments at baseline.”

 Methods

 Risk of bias assessment: References needed for the checklists – “Study quality was assessed by two reviewers using the RCT and and cohort studies Critical Appraisal Skills Programme (CASP) checklists respectively.”

 The following section is unclear and needs further explanation – “We calculated the mean FMA-UE score for the missing participants at baseline and subtracted the mean FMA-UE baseline score for the stroke type in the study. Thereby a negative score indicates that more severe individuals were not followed-up and vice versa.”

 Data synthesis and analysis: Need to rephrase the sentence for clarity – “Descriptive statistics (SPSS version 27) were used to calculate data separately for ischaemic stroke and ICH, and statistical tests (Independent Samples T-Test for normally distributed data, Mann Whitney U-Test for non-normal data and Person’s Chi-square test for distribution) were conducted for differences in baseline characteristics and outcomes between the two groups (ischemic stroke and ICH).” Contents within the bracket needs to be presented in a running sentence format. Unclear if authors mean Pearson’s Chi-square test was used for checking the normal distribution? It might be good to use Shapiro wilk or Kolmogorov-Smirnov test to test the normality.

 Results: In the below statement authors state five out of seven studies were excluded giving an impression of two studies were retained. Could be re written for the clarity - “Data from seven from the above 19 studies were obtained, but five studies were excluded because they did not satisfy all eligibility criteria, resulting in the inclusion of three studies in the review.”

 Risk of bias: It would be good to define what is considered as moderate to low and what is considered as low in this statement – “The risk of bias (Supplementary Table 4 and 5) in the reported studies was moderate to low and moderate.” It would be good to provide some information on the domains assessed for ROB and the findings for each study in main text.

 Authors can consider the use of intention to treat analysis to make appropriate adjustments to the data of patients with attrition and making scientific judgement - “Rather, there were more IS patients that were lost to follow-up and the patients lost to follow-up were more severely affected than the mean sample in IS than ICH. Therefore, the difference between the groups that we observed was not over-estimated, if anything attrition could have led to the improvements in the ischaemic stroke group being over-estimated.”

 Characteristics of the studies: This statement can be removed as the FMA UE was the inclusion criteria – “All used the FMA-UE to measure upper limb motor function.”

 Table 1.:  It would be good to add follow up scores of FMA UE (mean, SD) and follow up sample size in Table 1 along with the duration of follow up in each study. In addition, providing mean differences observed in each group over the time would add value. Figure 2. Should be presented before the discussion section to maintain the logical flow of the content.

 Discussion: It’s unclear how current study informs the choice of life saving early interventions. It would be beneficial to explain further before making the following conclusions “Conclusive evidence of recovery differences between stroke types would be important because in addition to informing patient and carer conversations about prognosis they are vital to inform appropriate care decisions. These include lifesaving early interventions of intensive care, neurosurgical interventions and specialist rehabilitation to optimise recovery.”

 In the discussion, authors state there were significant drop outs in the ischemic stroke group and that resulted in amplifying the mean improvements in the ischemic group stroke. It would be good to consider the use of intention to treat analysis to make the adjustments and draw scientific conclusions on the data.

 Grammatical errors seen in the following sentences: “..the 30% of survivors who do not follow the proportional rule and do not recovery arm function.”; “Individuals with ICH appear to recover more than those with ischaemic stroke since they started from lower levels of upper limb motor function and individuals with severe impairment do recover”; “A number of factors influence recovery after stroke including ages, acute care and stroke severity which are difficult to control…..”

Are the rationale for, and objectives of, the Systematic Review clearly stated?

Partly

Is the statistical analysis and its interpretation appropriate?

Yes

Are sufficient details of the methods and analysis provided to allow replication by others?

Yes

Are the conclusions drawn adequately supported by the results presented in the review?

Yes

Reviewer Expertise:

Stroke rehabilitation, physical activity, behaviour change, mhealth

I confirm that I have read this submission and believe that I have an appropriate level of expertise to confirm that it is of an acceptable scientific standard, however I have significant reservations, as outlined above.

# Response

**Published:** 2025-02-06

**Authors:** Ulrike Hammerbeck

**Affiliations:** Centre for Human and Applied Physiological Science, King's College London, London, England, UK

Dear Reviewer Thank you for taking the time to review this paper. Please see itemised responses to your remarks.

 The manuscript addresses a significant gap in the literature and is well written. This systematic review follows PRISMA guidelines and summarises the literature on motor recovery in ischemic stroke and intracerebral haemorrhage. There is logical reporting throughout the manuscript which makes it easy to read. To enhance clarity, some components of the manuscript could be further expanded.

 Title reads as: Comparing motor recovery in ischemic stroke and

 intracerebral haemorrhage: A Systematic Review. In the title, authors might want to be specific and consider the addition of ‘upper limb’ motor recovery and stage of stroke recovery of the chosen population (acute and subacute), as well as the chosen outcome measure (FMA UE), as the review focused only on upper extremity motor recovery and is very specific to the outcome measure used i.e.  FMA (other outcome measures of motor recovery were not included) and focused on acute and subacute stroke (excluded adults with chronic stroke).

**Author response:** *We have added the motor recovery domain of the upper limb and the time point after stroke. We have termed it subacute stroke as acute is often seen as the first 2 weeks after stroke which this paper does not investigate. The authors believe that the Fugl-Meyer is a widely accepted measure of upper limb impairment and believe that it is very clear from the abstract and manuscript that this was used and therefore have not included it in the title.* *Comparing upper limb motor recovery in subacute ischemic stroke and intracerebral hemorrhage: A Systematic Review*

 Abstract: It might be good to add aim/objective of the review under the ‘background’ than stating the implications of the research. This line can be replaced with the aim of the review - “This information could inform discussions with patients about their recovery prognosis as well as identify appropriate rehabilitation settings.”

**Author response:** *The authors feel that the previous sentence can be turned into a statement from a current question and thereby fulfil the purpose of stating the aim without changes to the word count. We have rephrased to: This paper aims to establish whether their respective upper limb motor impairment and recovery differ.*

 Introduction: Introduction looks brief. It would be good to give some background information/ literature available on the upper limb recovery in people with ischaemic and intracerebral haemorrhagic stroke under introduction. It is good to state the aim of the review in the last part of the introduction.

**Author response:** *We have added a paragraph on upper limb recovery and changed the wording to aim at the end of the paragraph.* *Arm weakness impacts on stroke survivors’ independence, quality of life and ability to maintain previous life roles 7,8. Despite significant recent advances in stroke care, over 20% of stroke survivors do not recover functional use of their arm 3. The impact of poor arm recovery is considerable with an estimated 100,000 strokes occurring in the UK every year, and 1.2 million stroke survivors alive at any time 9. However, longitudinal epidemiological studies of stroke recovery are very rare 10 and the natural history of recovery after stroke not well understood, especially in ICH. Arm recovery is influenced by patient demographics, stroke severity and acute care 11,12. However, it is currently unclear whether upper limb impairment and recovery differs between ICH and ischaemic stroke..* *We here aim to establish whether there is evidence of differences in upper limb motor recovery between ischaemic stroke and ICH by performing a systematic review of the literature.*

 It’s unclear how the following statement relates with the current study, as the current study examines the motor recovery post stroke. Authors might want to link the statement with the present study with the use of evidence - “Currently, negativity of ICH outcome prevails, and clinicians do not instigate lifesaving interventions including admission to intensive care and neurosurgical evacuation of the haematoma when patients with ICH present with severe impairments at baseline.”

**Author response:** *We have clarified this statement. The knowledge will shape goal setting and discharge planning. Furthermore, this information could assist clinical decision-making with regards to life saving interventions early after stroke and rehabilitation pathways at later time points*

 Methods

 Risk of bias assessment: References needed for the checklists – “Study quality was assessed by two reviewers using the RCT and and cohort studies Critical Appraisal Skills Programme (CASP) checklists respectively.”

**Author response:** *Included*

 The following section is unclear and needs further explanation – “We calculated the mean FMA-UE score for the missing participants at baseline and subtracted the mean FMA-UE baseline score for the stroke type in the study. Thereby a negative score indicates that more severe individuals were not followed-up and vice versa.”

**Author response:** *This section now reads: To established whether attrition caused a risk of bias through missingness of more severely affected individuals. To do this we calculated the mean FMA-UE score for the missing participants at baseline and subtracted the mean FMA-UE baseline score for the stroke type in the study. Thereby a negative score indicates that more severe individuals were missing for followed-up for the respective stroke type.*

 Data synthesis and analysis: Need to rephrase the sentence for clarity – “Descriptive statistics (SPSS version 27) were used to calculate data separately for ischaemic stroke and ICH, and statistical tests (Independent Samples T-Test for normally distributed data, Mann Whitney U-Test for non-normal data and Person’s Chi-square test for distribution) were conducted for differences in baseline characteristics and outcomes between the two groups (ischemic stroke and ICH).” Contents within the bracket needs to be presented in a running sentence format. *Done* Unclear if authors mean Pearson’s Chi-square test was used for checking the normal distribution? It might be good to use Shapiro wilk or Kolmogorov-Smirnov test to test the normality.

**Author response:** *The use of the Pearson’s chi-square test as a measure to investigate categorical distribution has been expanded upon. The section now reads: Descriptive statistics, using SPSS version 27, were used to calculate data separately for ischaemic stroke and ICH.  Statistical tests to establish differences in baseline characteristics and outcomes between stroke types used independent samples t-test for normally distributed data, Mann Whitney U-test for non-normal data and Pearson’s chi-square test to investigate categorical distribution.*

 Results: In the below statement authors state five out of seven studies were excluded giving an impression of two studies were retained. Could be re written for the clarity - “Data from seven from the above 19 studies were obtained, but five studies were excluded because they did not satisfy all eligibility criteria, resulting in the inclusion of three studies in the review.”

**Author response:** *Done*

 Risk of bias: It would be good to define what is considered as moderate to low and what is considered as low in this statement – “The risk of bias (Supplementary Table 4 and 5) in the reported studies was moderate to low and moderate.” It would be good to provide some information on the domains assessed for ROB and the findings for each study in main text.

**Author response:** *We have clarified in the text that the degree of the risk of bias has been reported for the respective studies as indicated by the references. We have added the description of ROB.* *We believe that we have outlined the key ROB factors in the paragraph and reference the relevant papers where this was the case. However, for further clarity we have restructured some sentences and added further detail.*

 Authors can consider the use of intention to treat analysis to make appropriate adjustments to the data of patients with attrition and making scientific judgement - “Rather, there were more IS patients that were lost to follow-up and the patients lost to follow-up were more severely affected than the mean sample in IS than ICH. Therefore, the difference between the groups that we observed was not over-estimated, if anything attrition could have led to the improvements in the ischaemic stroke group being over-estimated.”

**Author response:** *We report on studies that investigated outcome after either ICH or ischemic stroke. It appears as though the studies did indeed use an intention to treat analysis for their respective analyses.  However, the two health conditions are quite different, and ICH has far worse health outcomes. Therefore, there is a danger of Risk of Bias, and overestimation of effect, introduced by attrition in this population with an intention to treat analysis. We however established that attrition did not introduce a risk of bias and thereby an overestimation of effect.*

 Characteristics of the studies: This statement can be removed as the FMA UE was the inclusion criteria – “All used the FMA-UE to measure upper limb motor function.” *Removed.* Table 1.:  It would be good to add follow up scores of FMA UE (mean, SD) and follow up sample size in Table 1 along with the duration of follow up in each study. In addition, providing mean differences observed in each group over the time would add value.

**Author response:** *We have already included this information in the supplementary Table 6. We have included signposting to this data in the text and believe that due to the complexity of timepoints it is better to present in its entirety here. We have changed the order of the supplementary tables accordingly.*

 Figure 2. Should be presented before the discussion section to maintain the logical flow of the content.

**Author response:** *Formatting is done by the journal and we don’t have any concerns regarding this.*

 Discussion: It’s unclear how current study informs the choice of life saving early interventions. It would be beneficial to explain further before making the following conclusions “Conclusive evidence of recovery differences between stroke types would be important because in addition to informing patient and carer conversations about prognosis they are vital to inform appropriate care decisions. These include lifesaving early interventions of intensive care, neurosurgical interventions and specialist rehabilitation to optimise recovery.”

**Author response:** *This has been clarified and now reads: ICH survivors are far more likely to be placed on a palliative care pathway than ischaemic stroke survivors, irrespective of their baseline severity or other prognostic factors 12. Thereby they miss out on lifesaving early interventions including intensive care, neurosurgical interventions and specialist rehabilitation to optimise recovery. Evidence of the recovery potential of ICH survivors should be integrated into prognostic models that inform early medical decision making.*

 In the discussion, authors state there were significant drop outs in the ischemic stroke group and that resulted in amplifying the mean improvements in the ischemic group stroke. It would be good to consider the use of intention to treat analysis to make the adjustments and draw scientific conclusions on the data.

**Author response:** *The scientific data are drawn from the analysis using intention to treat but the ROB assessment showed that there was not an overestimation of the effect of ICH.*

 Grammatical errors seen in the following sentences: “..the 30% of survivors who do not follow the proportional rule and do not recovery arm function.”; “Individuals with ICH appear to recover more than those with ischaemic stroke since they started from lower levels of upper limb motor function and individuals with severe impairment do recover”; “A number of factors influence recovery after stroke including ages, acute care and stroke severity which are difficult to control…..”

**Author response:** *Addressed all these in the text.*

# Response

**Published:** 2025-02-13

**Authors:** Ulrike Hammerbeck

**Affiliations:** Centre for Human and Applied Physiological Science, King's College London, London, England, UK

Dear Reviewer Thank you for taking the time to review this paper. Please see itemised responses to your remarks.

**Comments:** The manuscript addresses a significant gap in the literature and is well written. This systematic review follows PRISMA guidelines and summarises the literature on motor recovery in ischemic stroke and intracerebral haemorrhage. There is logical reporting throughout the manuscript which makes it easy to read. To enhance clarity, some components of the manuscript could be further expanded.

 Title reads as: Comparing motor recovery in ischaemic stroke and

 intracerebral haemorrhage: A Systematic Review. In the title, authors might want to be specific and consider the addition of ‘upper limb’ motor recovery and stage of stroke recovery of the chosen population (acute and subacute), as well as the chosen outcome measure (FMA UE), as the review focused only on upper extremity motor recovery and is very specific to the outcome measure used i.e.  FMA (other outcome measures of motor recovery were not included) and focused on acute and subacute stroke (excluded adults with chronic stroke).

**Author response:** *We have added the motor recovery domain of the upper limb and the time point after stroke. We have termed it subacute stroke as acute is often seen as the first 2 weeks after stroke which this paper does not investigate. The authors believe that the Fugl-Meyer is a widely accepted measure of upper limb impairment and believe that it is very clear from the abstract and manuscript that this was used and therefore have not included it in the title.* *Comparing upper limb motor recovery in subacute ischaemic stroke and intracerebral haemorrhage: A Systematic Review*

**Comments: **Abstract: It might be good to add aim/objective of the review under the ‘background’ than stating the implications of the research. This line can be replaced with the aim of the review - “This information could inform discussions with patients about their recovery prognosis as well as identify appropriate rehabilitation settings.

**Author response: **” *The authors feel that the previous sentence can be turned into a statement from a current question and thereby fulfil the purpose of stating the aim without changes to the word count. We have rephrased to: This paper aims to establish whether their respective upper limb motor impairment and recovery differ.*

**Comments:** Introduction: Introduction looks brief. It would be good to give some background information/ literature available on the upper limb recovery in people with ischaemic and intracerebral haemorrhagic stroke under introduction. It is good to state the aim of the review in the last part of the introduction.

**Author response:** *We have added a paragraph on upper limb recovery and changed the wording to aim at the end of the paragraph.* *Arm weakness impacts on stroke survivors’ independence, quality of life and ability to maintain previous life roles 7,8. Despite significant recent advances in stroke care, over 20% of stroke survivors do not recover functional use of their arm 3. The impact of poor arm recovery is considerable with an estimated 100,000 strokes occurring in the UK every year, and 1.2 million stroke survivors alive at any time 9. However, longitudinal epidemiological studies of stroke recovery are very rare 10 and the natural history of recovery after stroke not well understood, especially in ICH. Arm recovery is influenced by patient demographics, stroke severity and acute care 11,12. However, it is currently unclear whether upper limb impairment and recovery differs between ICH and ischaemic stroke.* *We here aim to establish whether there is evidence of differences in upper limb motor recovery between ischaemic stroke and ICH by performing a systematic review of the literature.*

**Comments:** It’s unclear how the following statement relates with the current study, as the current study examines the motor recovery post stroke. Authors might want to link the statement with the present study with the use of evidence - “Currently, negativity of ICH outcome prevails, and clinicians do not instigate lifesaving interventions including admission to intensive care and neurosurgical evacuation of the haematoma when patients with ICH present with severe impairments at baseline.

**Author response: **” *We have clarified this statement. The knowledge will shape goal setting and discharge planning. Furthermore, this information could assist clinical decision-making with regards to life saving interventions early after stroke and rehabilitation pathways at later time points*

**Comments:**

 Methods

 Risk of bias assessment: References needed for the checklists – “Study quality was assessed by two reviewers using the RCT and and cohort studies Critical Appraisal Skills Programme (CASP) checklists respectively.” *Included*

 The following section is unclear and needs further explanation – “We calculated the mean FMA-UE score for the missing participants at baseline and subtracted the mean FMA-UE baseline score for the stroke type in the study. Thereby a negative score indicates that more severe individuals were not followed-up and vice versa.

**Author response: **” *This section now reads: To established whether attrition caused a risk of bias through missingness of more severely affected individuals. To do this we calculated the mean FMA-UE score for the missing participants at baseline and subtracted the mean FMA-UE baseline score for the stroke type in the study. Thereby a negative score indicates that more severe individuals were missing for followed-up for the respective stroke type.*

**Comments: **Data synthesis and analysis: Need to rephrase the sentence for clarity – “Descriptive statistics (SPSS version 27) were used to calculate data separately for ischaemic stroke and ICH, and statistical tests (Independent Samples T-Test for normally distributed data, Mann Whitney U-Test for non-normal data and Person’s Chi-square test for distribution) were conducted for differences in baseline characteristics and outcomes between the two groups (ischemic stroke and ICH).” Contents within the bracket needs to be presented in a running sentence format. *Done* Unclear if authors mean Pearson’s Chi-square test was used for checking the normal distribution? It might be good to use Shapiro wilk or Kolmogorov-Smirnov test to test the normality.

**Author response:** *The use of the Pearson’s chi-square test as a measure to investigate categorical distribution has been expanded upon. The section now reads: Descriptive statistics, using SPSS version 27, were used to calculate data separately for ischaemic stroke and ICH.  Statistical tests to establish differences in baseline characteristics and outcomes between stroke types used independent samples t-test for normally distributed data, Mann Whitney U-test for non-normal data and Pearson’s chi-square test to investigate categorical distribution.*

**Comments:**

 Results: In the below statement authors state five out of seven studies were excluded giving an impression of two studies were retained. Could be re written for the clarity - “Data from seven from the above 19 studies were obtained, but five studies were excluded because they did not satisfy all eligibility criteria, resulting in the inclusion of three studies in the review.”

**Author response:** *Done*

**Comments:** Risk of bias: It would be good to define what is considered as moderate to low and what is considered as low in this statement – “The risk of bias (Supplementary Table 4 and 5) in the reported studies was moderate to low and moderate.” It would be good to provide some information on the domains assessed for ROB and the findings for each study in main text.

**Author response:** *We have clarified in the text that the degree of the risk of bias has been reported for the respective studies as indicated by the references. We have added the description of ROB.* *We believe that we have outlined the key ROB factors in the paragraph and reference the relevant papers where this was the case. However, for further clarity we have restructured some sentences and added further detail.*

**Comments:** Authors can consider the use of intention to treat analysis to make appropriate adjustments to the data of patients with attrition and making scientific judgement - “Rather, there were more IS patients that were lost to follow-up and the patients lost to follow-up were more severely affected than the mean sample in IS than ICH. Therefore, the difference between the groups that we observed was not over-estimated, if anything attrition could have led to the improvements in the ischaemic stroke group being over-estimated.”

**Author response:** *We report on studies that investigated outcome after either ICH or ischemic stroke. It appears as though the studies did indeed use an intention to treat analysis for their respective analyses.  However, the two health conditions are quite different, and ICH has far worse health outcomes. Therefore, there is a danger of Risk of Bias, and overestimation of effect, introduced by attrition in this population with an intention to treat analysis. We however established that attrition did not introduce a risk of bias and thereby an overestimation of effect.*

**Comments:** Characteristics of the studies: This statement can be removed as the FMA UE was the inclusion criteria – “All used the FMA-UE to measure upper limb motor function.” *Removed.* Table 1.:  It would be good to add follow up scores of FMA UE (mean, SD) and follow up sample size in Table 1 along with the duration of follow up in each study. In addition, providing mean differences observed in each group over the time would add value.

**Author response:** *We have already included this information in the supplementary Table 6. We have included signposting to this data in the text and believe that due to the complexity of timepoints it is better to present in its entirety here. We have changed the order of the supplementary tables accordingly.*

**Comments: **Figure 2. Should be presented before the discussion section to maintain the logical flow of the content.

**Author response:** *Formatting is done by the journal and we don’t have any concerns regarding this.*

**Comments:** Discussion: It’s unclear how current study informs the choice of life saving early interventions. It would be beneficial to explain further before making the following conclusions “Conclusive evidence of recovery differences between stroke types would be important because in addition to informing patient and carer conversations about prognosis they are vital to inform appropriate care decisions. These include lifesaving early interventions of intensive care, neurosurgical interventions and specialist rehabilitation to optimise recovery.”

**Author response:** *This has been clarified and now reads: ICH survivors are far more likely to be placed on a palliative care pathway than ischaemic stroke survivors, irrespective of their baseline severity or other prognostic factors 12. Thereby they miss out on lifesaving early interventions including intensive care, neurosurgical interventions and specialist rehabilitation to optimise recovery. Evidence of the recovery potential of ICH survivors should be integrated into prognostic models that inform early medical decision making.*

**Comments: **In the discussion, authors state there were significant drop outs in the ischemic stroke group and that resulted in amplifying the mean improvements in the ischemic group stroke. It would be good to consider the use of intention to treat analysis to make the adjustments and draw scientific conclusions on the data.

**Author response:** *The scientific data are drawn from the analysis using intention to treat but the ROB assessment showed that there was not an overestimation of the effect of ICH.*

**Comments: **Grammatical errors seen in the following sentences: “..the 30% of survivors who do not follow the proportional rule and do not recovery arm function.”; “Individuals with ICH appear to recover more than those with ischaemic stroke since they started from lower levels of upper limb motor function and individuals with severe impairment do recover”; “A number of factors influence recovery after stroke including ages, acute care and stroke severity which are difficult to control…..”

**Author response:** *Addressed all these in the text.*

# Reviewer report: Reviewer response for version 1

**DOI:** 10.21956/healthopenres.14537.r27655

**Published:** 2023-11-27

**Authors:** Margit Alt Murphy

**Affiliations:** 1 Clinical Neuroscience, University of Gothenburg, Gothenburg, Västra Götaland County, Sweden; 2 Sahlgrenska University Hospital, Gothenburg, Sweden

The manuscript is well written and targets an important and timely issue. Taken the current changes in stroke care and rehabilitation, the understanding of motor recovery pattern in haemorrhagic strokes is important, and needed. The results of this study can guide stroke rehabilitation and improve adaptive selection of interventions. All parts of the manuscript are carefully and methodically reported, which makes the reading easy. The results are clearly stated, limitations pointed out and conclusions relevant and well inside the study research question.

 Taken that only 3 studies could be included, and all were from similar regional context, makes the results less applicable for a wider geographical spread. But, this is acknowledged by the authors, but maybe could be pointed out even more clearly.

 The authors have probably made a thorough search of the topic, and it seems that the evidence is limited. The limited number of studies is also a result of quite specific inclusion criteria used. So maybe some more discussion on that, and if there are other studies that could have been included if other outcome measures would have been allowed (ARAT, NIHS arm). It would also be nice to add some information on research base and evidence regarding motor recovery in general and not only upper limb. In summary, the manuscript could be indexed as it is, but it would be even more interesting if the discussion could include a somewhat wider perspective beyond the upper limb and Fugl-Meyer outcome.

Are the rationale for, and objectives of, the Systematic Review clearly stated?

Yes

Is the statistical analysis and its interpretation appropriate?

Yes

Are sufficient details of the methods and analysis provided to allow replication by others?

Yes

Are the conclusions drawn adequately supported by the results presented in the review?

Yes

Reviewer Expertise:

Upper limb motor rehabilitation in stroke

I confirm that I have read this submission and believe that I have an appropriate level of expertise to confirm that it is of an acceptable scientific standard.
